# Data-Driven Strain Sensor Design Based on a Knowledge Graph Framework

**DOI:** 10.3390/s24175484

**Published:** 2024-08-24

**Authors:** Junmin Ke, Furong Liu, Guofeng Xu, Ming Liu

**Affiliations:** 1 Key Laboratory of Trans-Scale Laser Manufacturing, Beijing University of Technology, Ministry of Education, Beijing 100124, Chinaliuming123@emails.bjut.edu.cn (M.L.); 2 School of Physics and Optoelectronic Engineering, Beijing University of Technology, Beijing 100124, China

**Keywords:** knowledge graph, strain sensor, machine learning, material science

## Abstract

Wearable flexible strain sensors require different performance depending on the application scenario. However, developing strain sensors based solely on experiments is time-consuming and often produces suboptimal results. This study utilized sensor knowledge to reduce knowledge redundancy and explore designs. A framework combining knowledge graphs and graph representational learning methods was proposed to identify targeted performance, decipher hidden information, and discover new designs. Unlike process-parameter-based machine learning methods, it used the relationship as semantic features to improve prediction precision (up to 0.81). Based on the proposed framework, a strain sensor was designed and tested, demonstrating a wide strain range (300%) and closely matching predicted performance. This predicted sensor performance outperforms similar materials. Overall, the present work is favorable to design constraints and paves the way for the long-awaited implementation of text-mining-based knowledge management for sensor systems, which will facilitate the intelligent sensor design process.

## 1. Introduction

Due to recent advancements in nanomaterials and nanotechnology, wearable electronic devices, particularly flexible and stretchable strain sensors, have garnered significant interest across various fields, such as healthcare and human motion detection, sports and physical training, and smart robotics [1,2]. In this process, these devices can be directly attached to the body to collect signal information, which can be utilized for various physical activities and health states. Currently, 5G communication technology has matured, and strain sensors are anticipated to have extensive applications in the field of motion perception systems.

Generally, a reliable implementation for detecting strain signals involves hybridizing a network composed of conductive materials (metal nanowires, carbon-based materials, etc.) with stretchable substrates (cotton, yarn, etc.). The indicators of strain sensor performance include gauge factor, strain range, long-term stability, and response time. The gauge factor and strain range are the two most critical factors among them, as they have a direct correlation with the application scenarios of strain sensors [3]. The performance of a strain sensor is significantly influenced by the choice of materials and their respective structures. Currently, viable research is divided into two categories. The first category involves designing sensor structures, such as segregated conductive networks and polymeric conductive composites [4,5,6,7]. The second category focuses on incorporating advanced nanomaterials such as 0D carbon black, metal nanoparticles, and graphene, as well as flexible substrates such as rubber [8,9,10,11,12] to improve sensors’ performance. For the design of strain sensors, most of the work to date has been focused on improving performance with a high sensitivity, strain range, and cycle ability, etc. This leads to extensive trial-and-error experiments to optimize the process parameters, which involves high costs. The data-driven method is no doubt a new research paradigm for designing high-performance strain sensors, which have already been used in some fields. Recent research has concentrated on incorporating machine learning techniques (ML) into sensor design processes to forecast sensor characteristics [13,14,15,16,17,18,19,20]. Wang et al. [21] developed a silent speech recognition system based on a linear discriminant analysis (LDA) algorithm. Ravenscroft et al. [18] implemented a selection of machine learning algorithms, including artificial neural networks (ANN), random forests (RF), and k-nearest neighbor (KNN), for categorizing input signals. The above studies rely on ML methods to recognize the motion information. In particular, using ML techniques to facilitate the sensor design process has seldom been investigated. An alternative method involves mathematical statistics or sensor modeling tools to optimize sensor characteristics [22]. Remarkably, a recent study utilized active learning technology to automate the design of sensors based on composites [23]. Although these methods have their advantages, the intricate relationships within the sensor data and the influence of various semantic information on the performance are still unclear. Sensor design may consider factors of conductive materials, flexible substrates, manufacturing methods, and structures to optimize performance. The correlation among the above items, which are regarded as semantic features in the ML field, is crucial for discovering the underlying information. However, utilizing semantic features, especially the relationship feature, into a single regression model (based on text feature) poses a significant challenge in facilitating sensor designs. Moreover, current predictions usually have a unidirectional nature, concentrating exclusively on either the positive or negative aspects of the design process. Consequently, the current ML regression methods lack the ability to describe sensor knowledge.

Therefore, there is a need to develop a new framework that harnesses machine learning techniques for accurate predictions and recommendations based on semantic features while efficiently managing sensor knowledge, particularly in the era of rapid data growth. Effective knowledge interpretation necessitates a thorough understanding of the sensor. In this context, knowledge graphs (KGs) can facilitate the creation of unified standard representations for data fusion by representing knowledge in the form of entities and relationships. It can aggregate the relationships of multiple aspects via semantic associations. By extracting semantic features, it can serve as a foundation for the development of robust modeling. Despite their effectiveness in managing and visualizing complex relationships and characteristics in the literature [24,25], knowledge graphs are still underutilized for innovative design discovery. Since representational learning methods can extract multi-dimensional information as inputs, it has been proposed to apply representational learning methods to deep feature processing for prediction and the discovery of new things. For example, Nie et al. utilized word embeddings to extract semantics features for literature mining of materials [25]. However, the disadvantage of this method is that the extracted features lack structural information, which is difficult to apply to the task of sensors. Therefore, a machine learning framework based on the combination of knowledge graphs and representational learning was further developed for the prediction of strain sensors. This framework’s efficiency is described as follows. (1) Storing both textual and data information in an organized manner and using algorithms to convert it into scientific knowledge. (2) Efficiently conducting reasoning analyses on a trained neural network after learning its representation, which involves tasks such as mining sensor designs and predicting performance. (3) The finding design is relatively consistent with the model and shows excellent sensing performance (300% of strain range). Meanwhile, the durability of the sensor (above 1000 cycles under 10% strain) is excellent under the stretching–releasing process. Compared to extreme strain cases (small stain range (<50%) or low sensitivity (<20)), the performance enables real-time monitoring of in-plane strains between 30% and 300%. (4) Ultimately, once established, the AI framework becomes accessible, as scientific knowledge from previous publications can serve as a guide or platform for the rapid design of multifunctional devices in the future.

## 2. Materials and Methods

**Figure 1:**
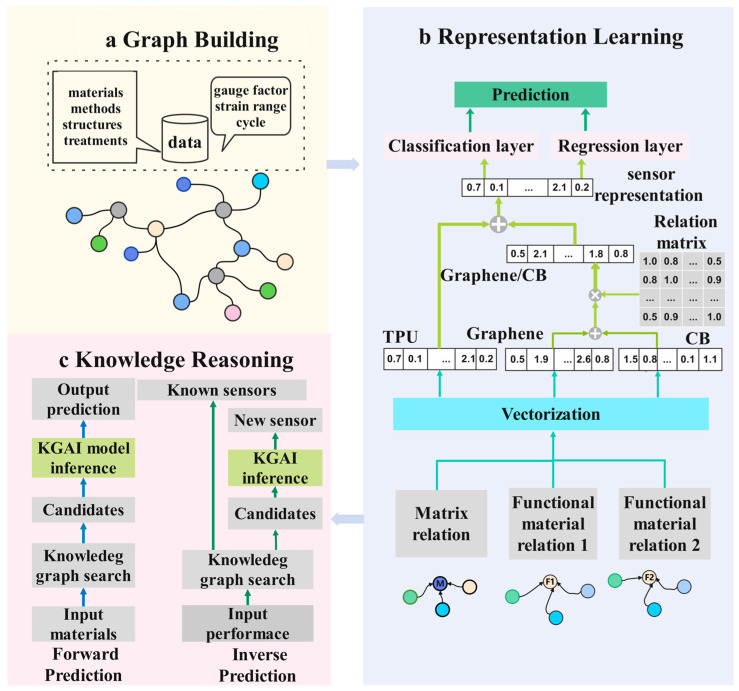
Illustration of a KGAI architecture developed with three procedures for (**a**) knowledge graph building of strain sensors, (**b**) representation learning of the knowledge graph (feature engineering), (**c**) knowledge reasoning of sensor design (performance prediction, sensor design query, and exploration).

The purpose of this work is to create a new framework (KGAI) based on the knowledge graph for properties prediction and design exploration. Graph construction, representation learning, and knowledge reasoning are the three primary modules of the KGAI architecture (Figure 1) in the context of data-driven AI analyses.

### 2.1. Knowledge Graph Building of the Strain Sensors

Firstly, 200 fabrication methods of strain sensors based on piezoresistive mechanisms were collected from the literature. Secondly, the nodes of the graph were extracted by keyword-matching rules and manual extraction. These nodes include materials (flexible and conductive), manufacturing methods, sensor structure, and performance (strain range, measurement factor, and period). Among them, flexible materials included cotton, ecoflex, thermoplastic polyurethane (TPU), fibers, polymethylsiloxane (PDMS), nylon, polyurethane, rubber, and fabrics. Conductive materials included carbon nanotubes (CNTs), MXene, carbon black (CB), graphene, gold nanoparticles (AuNPs), silver nanowires (AgNWs), reduced graphene oxide (rGO), multi-carbon nanotubes (MWCNTs), PEDOT: PSS, cellulose nanofibers (CNFs), polyvinylidene fluoride (PVDF), cellulose nanocrystals (CNCs), carbon nanoparticles, polyacrylonitrile, and liquid metals. Then, the node data were standardized (SS1). After that, the triples were built using the above nodes as entities (Figure S2). In a triple, there is a head node, a relationship node, and a tail node, where the relationship between the head and tail nodes can be learned. The relationship can be divided into the following aspects: flexible matrix, functional material, structure, method, strain range, measurement factor, and cyclic stability. The number of constructed triples and entities is 978 and 2000, respectively. Finally, all triples were written to the graph database (nebula graph database). 

### 2.2. Representation Learning 

The representative learning module in Figure 1b converts semantic relationships from text strings into low-dimensional dense vectors, quantifying data within the knowledge graph using matrix calculations. Generally, generating embedding is a key procedure that affects the overall performance of the method. Given the size of the database, four lightweight algorithms (TranSE [26], DistMult [27], HOLE [28], and Node2vec [29]) are utilized. Meanwhile, two general indicators of Mean Reciprocal Rank (MRR) and Hit@n (namely, the proportion of correct entities ranked in the top 1/3/5/10) [26,27,28] are then applied to compare the performance of the algorithms above. The specific definitions of these two factors are given in detail in the Supplementary Materials. The aim is to learn representations of entities and relations that best explain a dataset by minimizing the logistic loss. The training epoch is 10,000. The optimizer is stochastic gradient descent, and the learning rate is 0.01. The L2_regularization is used for avoiding over-fitting and is set to 0.01.

### 2.3. Knowledge Reasoning

In the last stage of knowledge reasoning, functions such as prediction, mining, and detection are enabled using the embedding vectors established in the previous module. To achieve the property prediction and new findings, machine learning techniques that learn from a training dataset are utilized to make accurate predictions on unseen samples. Three robust and nonlinear methods of extreme gradient boosting (XGB), support vector machine (SVM), and multi-layer perception (MLP) are employed to compare performance and identify the best model for determining the probability of combinations of flexible substrates and functional materials. Furthermore, to make a distinction between positive and false designs, the probability threshold of classification is determined after extracting the embedding of the entity. The classification threshold is set above 0.74, according to the probability range of positive material combinations of sensors in the literature (Table S2). 

### 2.4. Fabrication of a Designed Strain Sensor 

According to the knowledge reasoning, the TPU/graphene/CB system was first found with a high correlation (0.93). Meanwhile, TPU with conductive composites had good merit in balancing the sensitivity and strain range as compared to the traditional design [6,15,16,30,31,32,33] of PDMS, rubber, cotton, etc./graphene/CB; therefore, it was chosen in the present study. Subsequently, a commonly used electro-spinning method was used to create the TPU composite film.

First, 2 g of TPU was dissolved in a dimethylformamide (DMF)/tetrahydrofuran (THF) (Macklin, Shanghai, China) mixture solution (volume ratio of 1:1) for 6 h by magnetic stirring to form a 20 wt% solution. Subsequently, the collecting distance was set to 20 cm, a flow rate of 3 mL h^−1^, and a high voltage of 20 KV was supported. The syringe tip was 0.65 mm, and a rotation drum as the receiving device was set at 100 rpm. After 3 h, the TPU fibrous membranes were prepared. Next, 2 g of CB and graphene were ultrasonically dispersed in the solution (sodium dodecyl benzene sulfonate and deionized water) (Macklin, Shanghai, China) at 300 w ultrasonic power. Finally, the obtained TPU fibrous mats were placed in the suspension three times at the same work power. After three washing operations and drying at 70 °C for 90 min, the fibrous membrane was successfully prepared. The layer structure was obtained via the rolling-up process.

### 2.5. Characterization

The surface morphology of carbon-based TPU fibers was observed with a scanning electron microscope (JEOL 7001F, JEOL Ltd., Tokyo, Japan). Samples were subjected to electron microscopy after gold spraying. The electrical and mechanical properties of the fibers were measured by monitoring their resistance to the cyclic tensile tests in real-time with an electrochemical workstation (Ametek P4000, Ametek Inc., Berwyn, IL, USA) and Micro Tester (Instron 5948, Instron, Shanghai, China).

## 3. Results and Discussion

### 3.1. The Effect of the KGAI Representation Method 

**Figure 2:**
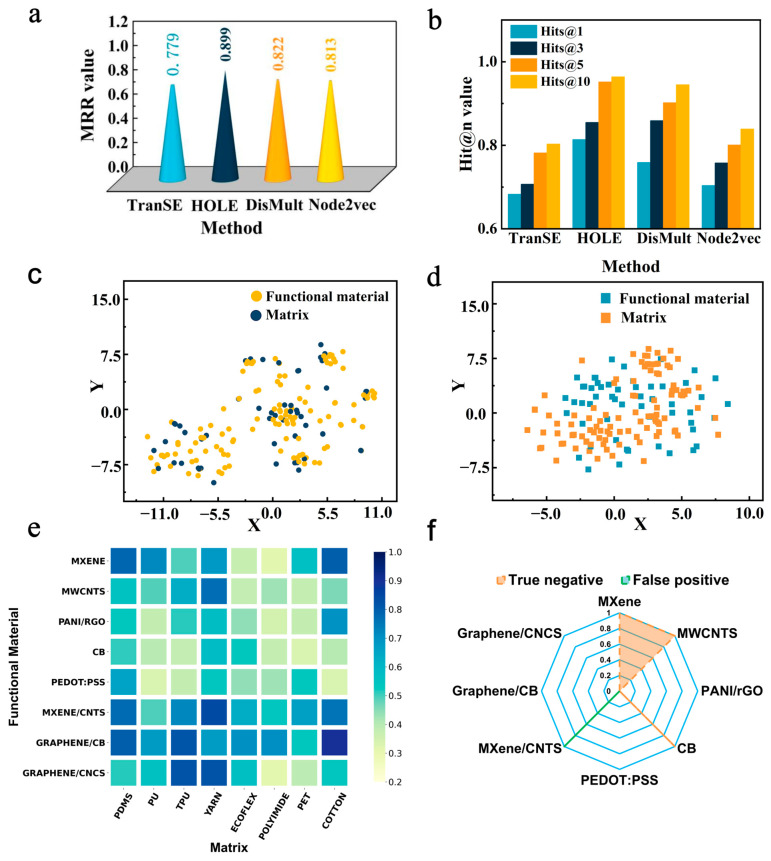
The evaluation of the KGAI method. (**a**,**b**) Values of MRR and Hit@n metrics of knowledge graph construction. (**c**,**d**) Cluster atlas of different combinations of representation methods (improved method with considering the correlation between functional materials and hole method without considering the correlation between functional materials) via unsupervised learning after dimensionality reduction. (**e**) The heat map of functional materials and flexible matrix. The colors scale with the values of the cosine similarity between embeddings. The dark color of the squares means a strong correlation. (**f**) The value of false positive (value = 1, wrong prediction in database) and true negative of designs (value = 3, undetected designs in database).

It is found that the algorithm of HOLE has the highest values of MRR (~0.899) and Hit@n (over 0.8) compared to the other three (Figure 2a,b). In order to consider the connections between multicomponent attributes, the same attribute relations are extracted through added embeddings, termed improved representations. For example, the combination of MXene and carbon nanotubes is examined both as a whole and as an individual, illuminating connections between various material combinations. Nevertheless, the original representation (OR) fails to take into account the relationship between materials.

To clarify the advantage of improved representation (IR), different combinations of flexible matrices and functional materials were tested. Figure 2c,d displays two-dimensional coordinates of various material combinations in the embedding space of the IR and OR methods. The distribution indicates that different functional materials with the same flexible substrate are more closely clustered together. The statement suggests that the design vector generated by the knowledge representation algorithm contains semantic information about sensor design. Additional findings from KMeans clustering indicate that a stronger correlation exists between flexible matrices and functional materials, leading to an increased likelihood of sensor design formation. Moreover, Table S3 provides a detailed list of typical combinations of matrix and functional material with a short distance from the improved method, which is consistent with previous experiments. Furthermore, the distance between the flexible substrate and functional materials in the new combinations is comparatively greater than in prior designs. The effective integration of existing material combinations between data indicates that the enhanced method is a reasonable representation of the sensor knowledge. Currently, the majority of studies [16,17,18,19,20] only focus on material properties as a feature to accomplish the prediction task. However, the relationship between materials in sensor design is vital, especially in the hydrogel design, which represents the interfacial property. 

A heat map, which was reported to extract the correlation among different vectors [23], was also applied to demonstrate the relevance of designs. The correlations between 8 flexible matrixes and 8 functional materials were then extracted, and the results are plotted in Figure 2e. The block’s color transition from pale yellow to dark yellow signifies a stronger correlation between them. Materials such as cotton, TPU, PDMS, and yarn exhibit significant correlations in their matrix structure, which is consistent with earlier research [34,35]. Previous research [36] has established a strong correlation between MXene and flexible matrices (PDMS and cotton). Furthermore, graphene/CB combinations with cotton and PDMS substrates follow the literature [37]. The observed correlation between TPU and graphene/CB is a new design due to comparable production processes. Despite the lack of direct reports for PEDOT/PSS combinations, the KGAI method’s correlation learning revealed potential for yarn and graphene/cellulose nano-crystalline (CNC) designs. The involvement of domain experts with a thorough understanding of the nuances and interactions of material factors is required to interpret these complexities. In conclusion, the information-to-knowledge route (KGAI method) is capable of searching for existing designs.

In order to evaluate the prediction error of the KGAI method, a standard radar graph [23] is presented in Figure 2f. The false positive stands for the number of wrong predictions of designs in the graph database, while the true negative represents undetected designs. The KGAI method demonstrates robust strain sensor design capabilities, with fewer than 4 wrong predictions out of 64 samples.

### 3.2. Navigation Model of Prediction

**Figure 3:**
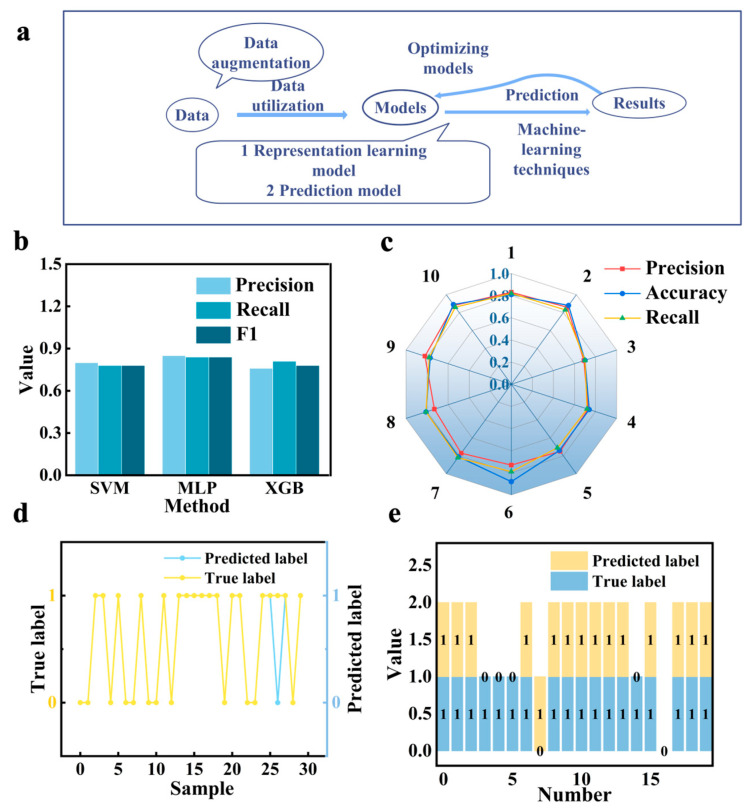
(**a**) The design process of the whole method. (**b**) The indicators (Precision, Accuracy, and Recall) of three methods for classification tasks by 10-fold cross-validation. (**c**) The indicators of the MLP method in ten different test datasets. (**d**,**e**) The predicted labels and true labels of train and test samples (one and four misclassified samples, respectively).

In order to assess prediction performance for the classification task (Figure 3a), typically used evaluation indicators like precision, recall, and F1-score were chosen. To evaluate the feasibility of the design, the classification task was executed. Ultimately, the multi-layer perceptron method performed better than SVM and XGB techniques and was selected as the study’s navigation model (Figure 3b). The MLP method’s accuracy and precision are shown in Figure 3c for ten distinct test sets, with corresponding values of 0.81 and 0.78, respectively. Figure 3d,e indicates that the predicted labels are basically consistent with the real labels.

**Figure 4:**
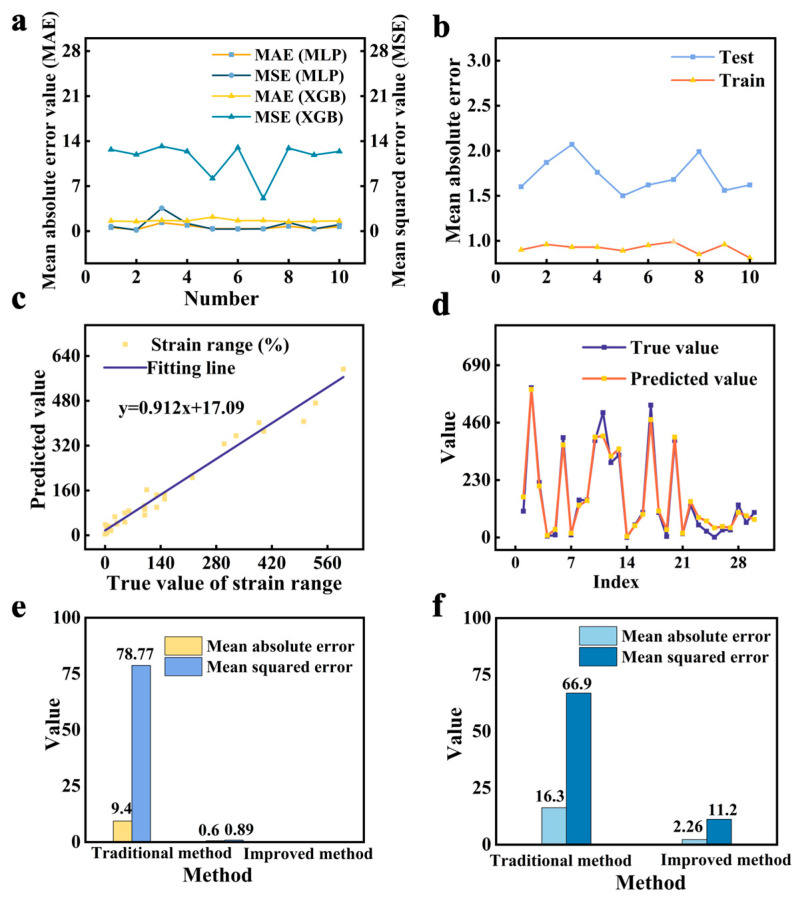
(**a**) The indicators of the XGB and MLP method for performance prediction. (**b**) The mean absolute error of 10-fold cross-validation of the test dataset. (**c**) The model prediction value and the true value for train samples. The line represents a prediction formula for the strain range. (**d**) The model prediction and the true value of test samples for strain range. (**e**,**f**) Evaluations of the design prediction. (**e**) The error prediction performance of the traditional method (only using the text feature without the correlation) and our KGAI method (using the graph feature containing the text feature and relationship feature) in the training process. (**f**) The error prediction performance of the traditional method and our KGAI method on the test dataset.

In this study, the prediction of performance is also considered and constructed, in addition to the possibility of predicting sensor designs. The regression task (XGB and MLP methods) utilizes the mean absolute error (MAE) and mean squared error (MSE) as measures of performance. Unlike accuracy, a smaller MAE value indicates a higher level of prediction accuracy in the decision-making process. Thus, the regression model is derived by training the multi-layer perception network using cross-validation to prevent over-fitting. The mean absolute error (MAE) is used as a metric to assess performance (Figure 4a,b). It is evident that the MAE and MSE of the MLP method are relatively smaller than SVM and XGB methods, owing to the learning and nonlinear approximation capabilities of the neural network. The final MAE of the MLP method on the training data is 0.98, and on the test data, it is 1.73. Additionally, the predictions of the data are illustrated in Figure 4c,d, showcasing relative fitting results. The results demonstrate that the feature generated by the relationship-based representation method is an effective way to predict properties.

To further demonstrate the advantages of the relationship-based representation method (KGAI), the traditional method directly using text features [24] (e.g., the word2vec method without considering relationships) is used to complete regression tasks for comparisons (partial data in Table S2). Figure 4e demonstrates that the KGAI method has reduced the maximum absolute value error by nearly 8 times and the maximum square value error by nearly 5 times in comparison to the traditional method (Figure 4f). The primary reason for the excellent results is the utilization of deeper-level information hidden in the data, enhancing representations of the interaction between materials. The findings suggest that using ergodic sensor attribute knowledge as an entity and using relationships between attributes as features can improve the precision of traditional text-based machine learning methods, hence facilitating strain sensor mining and design tasks. 

Most machine learning work of strain sensors in the literature either focuses on signal processing for resistance readout or lacks a comprehensive framework that combines knowledge graph-based data fusion with machine learning techniques for modeling. However, the graph embedding framework under study focuses on the correlation between performance and design, bridging the gap in the strain sensor field. This approach specifically addresses the issue of ignoring intra-aspect relationships, which is often present in medical modeling. The proposed method has obvious advantages: enhanced generalization, simplicity, and efficiency. The algorithm converts triples into low-dimensional and dense vectors to achieve efficient design reuse. A potential limitation of the present work is the inconsistent performance of most reports, restricting the capability of the machine learning model. Future data augmentation is warranted to enhance predictive power. This work is a preliminary demonstration of the application of the knowledge graph method for strain sensor fabrication. Future research could explore model optimization for the fabrication process.

### 3.3. Entity Prediction 

The knowledge reasoning process, as shown in Table 1, is capable of executing two-way tasks, including (1) sensor performance prediction and (2) recommendation of feasible sensor designs. Since the construction of a knowledge graph consists of key characteristics of sensors (referred to as entity), these two-way predictions can be regarded as entity predictions. The performance of strain sensors is evaluated by two crucial parameters: the gauge factor (GF) and strain range (l). GF is defined by the slope of the relative resistance change versus the strain range change. 

**Figure 5:**
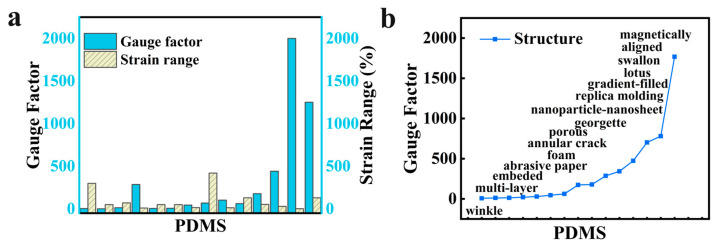
The trend of flexible substrate (PDMS) in the knowledge graph. (**a**) The sensor performance of strain range and gauge factor. (**b**) The trend of gauge factor under different structures of PDMS.

Table 2 presents a forward design for predicting performance based on the inputs of the flexible matrix and functional materials. The performance of rGO is significantly affected by the type of fabric it is coupled with, such as yarn and cotton, even if they are manufactured in a similar manner [38,39]. In an inverse design, Table 3 displays the recommended materials and structures for achieving an appropriate design with extremely high-performance results (e.g., 1 gauge factor, 120% strain range, and 500% strain range are set in advance). Moreover, the performance of various material combinations and structures can be captured. For example, Figure 5 demonstrates the trend of performance and structure strategies. Further, the balance between strain range and sensitivity of PDMS should be considered. In this regard, the utility of the KGAI framework enables rapid tracking of the current trend in strain sensors. 

### 3.4. Automatic Strain Sensor Design 

Due to the high correlation of materials in Section 3.1, TPU was first chosen as the matrix in this work due to its merits such as biocompatibility, breathability, flexibility, and toughness. The KGAI method found a mixed graphene/CB design due to their excellent correlation. The classification of this combination (TPU/graphene/CB) obtained a high probability (above 0.93), signifying the feasibility of this combination. Regarding the manufacturing method, electro-spinning technology was predicted to be used. The machine learning model applied to predict this combination had a good strain range (above 240%). Moreover, the verification in a later section shows that the combination of TPU and graphene/CB exhibits a strain range that is relatively consistent with the model prediction. 

### 3.5. Characteristics of the Designed Strain Sensor 

**Figure 6:**
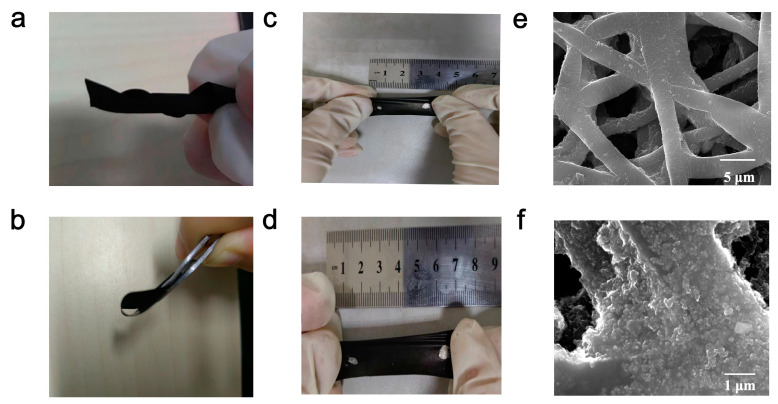
Photographs of the film showing excellent flexibility when (**a**) twisted, (**b**) bent, and (**c**,**d**) stretched. (**e**,**f**) Top views of the composite film under the SEM observation with different scales (5 μm and 1 μm).

The fabrication process of the strain sensor is shown in the Method section. Figure 6 demonstrates the characteristics of flexibility, ultra-thin structure, and surface morphology. The multi-layer network from the top surface of the film was observed. Additionally, it was found that carbon-based materials were evenly distributed on the fibers, attributed to the loose structure of the TPU substrate, which may facilitate good contact between functional materials and substrates, resulting in excellent stability [42].

**Figure 7:**
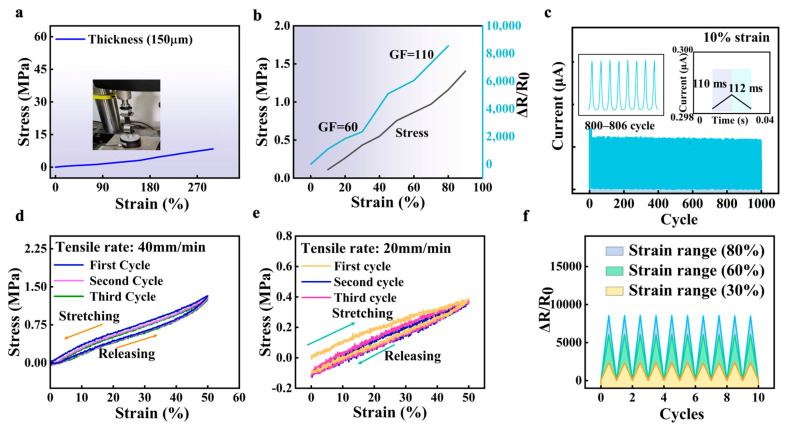
Main metrics of the strain sensor. (**a**) The stress under different strain ranges. (**b**) The relative resistance–strain variation curves and stress–strain curves of strain sensors. (**c**) The durability of the sensor. The insert pictures are the subgraph of the cycle (800–806) and response/recovery times of the sensor. (**d**,**e**) The three cycles of the stretching process with a tensile rate of 40 mm/min and 20 mm/min, respectively. (**f**) The cycle stability of relative resistances under different strains (35%, 45%, and 80%).

In order to verify practical performance, the mechanical properties of the strain sensor were first evaluated by a tensile test, as shown in Figure 7a. It is evident that over a 300% strain range was obtained, conforming to the prediction. Except for the strain range, the relative resistance change corresponding to the strain range is shown in Figure 7b, which demonstrated a good gauge factor (110 at 80% strain). When comparing spherical CB nanoparticles, CB and graphene are more likely to form a complete conductive network, thereby enhancing electrical conductivity. Table 4 summarizes the comparisons of performance (such as strain range, gauge factor, and durability) to compare the performance of the designed sensor. It can be seen that the designed sensor has a balanced performance between strain range (300%) and gauge factor (110 at 80% strain). The excellent performance can be attributed to its internal network structure. The fibers underwent straightening and elongating, leading to deformation of the internal network structure, resulting in resistance changes. Meanwhile, the micro-crack that is generated during the stretching process helps enhance the performance [21]. Figure 7c reveals the sensor ‘s excellent durability under the stretching–releasing process (10% of strain). The insert picture in Figure 7c shows the response time (110 ms) and recovery time (112 ms). To further demonstrate the stability of the strain sensor, different tensile rates (40 mm/min and 20 mm/min) were applied to the strain sensor, presenting repeatable stress (Figure 7d,e). The first cycle exhibits a relatively large variation in stress, most likely due to the permanent destruction of the conductive network, which aligns with the previously reported sensor [22]. Stress in subsequent cycles was reduced compared to the first cycle and almost overlapped, suggesting stable destruction and reconstruction of the conductive network. The response of the strain sensor during stretch–release cycles in various strain ranges (30%, 60%, and 80%) is depicted in Figure 7f. Different strains exhibit the same response patterns, suggesting a continuous reaction to cyclic loading. Furthermore, during the stretching and releasing process, the resistance may increase proportionally to the tensile stress and recover naturally without any external intervention. This phenomenon can be attributed to the partial breakdown of electrically conductive pathways and the gradual increase in separation between carbon-based materials, both leading to an increase in resistance. Furthermore, the sensing signals remain stable, and the alteration in stress is only determined by strain, indicating the reliability of the sensor in stress recognition.

### 3.6. Demonstration of a Human Action Monitoring Application

**Figure 8:**
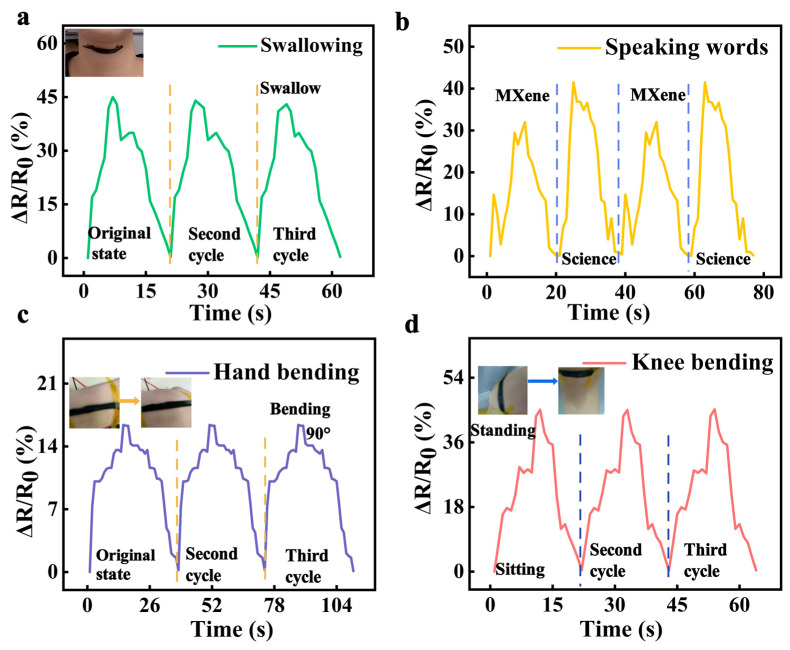
Applications of the strain sensor to detect human motions. (**a**,**b**) Motions of swallowing and speaking. (**c**) The resistance changes with the bending of the hand. (**d**) The resistance changes of knee motions (standing and sitting state).

According to the above results, the prepared composite sensor has excellent properties such as extensive strain range, flexibility, and good sensitivity, showing great prospects in wearable devices. Consequently, it was used to detect human motion across several levels, including swallowing, speaking, hand bending, and knee movements. The small strain of swallowing was first tested by attaching the sensor to the neck, followed by testing the speaking process of two words (“MXene” and “science”) (Figure 8a,b). Different changes in relative resistance during these activities caused by vocal cord vibrations were captured, indicating sensitivity to tiny motions. Figure 8c exhibits that the sensor responded to hand curvature with a consistent correlation between the bending angle and resistance change. To monitor larger joint movements, measurements were performed while sitting and standing by mounting the sensor on the knee (Figure 8d). The resistance-time waveform presented a clear and periodic response, showing promising potential for human-friendly rehabilitation. The pressure characteristic was also observed according to different forces (Figure S3).

As discussed above, the designed strain sensor has the potential for health applications. The high-quality design is mainly attributed to the constructed knowledge graph for strain sensors. On the surface, the sensor design data consists of discrete symbols that fulfill diverse requirements. However, these discrete symbols represent the semantic correlation between design elements, such as dimension mixtures. The relationship between designs (material, structure, property) of strain sensors is unveiled in the graph database, enabling data-driven mining. With more designs of films or sensors and appropriate data-processing methods, the model could be continuously updated, leading to improvement [45]. It is feasible to design multifunctional films, such as frost resistance, UV blocking, electromagnetic interference shielding, and so on.

## 4. Conclusions

This work develops a framework that combines knowledge graphs and graph representation learning methods to reveal the interconnectedness of strain sensor design. The established knowledge graph has a high MRR (mean reciprocal rank), with a value greater than 0.89, allowing for effective retrieval and querying of information. Furthermore, by establishing an MLP regression model, the feasibility of the design and strain range prediction are demonstrated. The strain sensor created using this framework has a prediction accuracy of 0.93, demonstrating its excellent performance (strain range of 300%) and the validity of this relational semantic prediction method in strain sensor design. The efficiency of this method not only opens up the possibility of rapid fabrication of strain sensors but it is also expected to extend to other material science design challenges by guiding algorithms to learn and perform specific tasks. For precise prediction, some promising directions could be considered in future work. (1) The current knowledge graph database could be expanded to encompass a wider variety of strain sensor data. This will involve the aggregation of data from diverse sources, thereby improving the model’s generalizability and applicability. Furthermore, the creation of industry-standard flexible sensor datasets is imperative to establish a benchmark. For example, the low detection limit, response time, biocompatibility, and cost should be properly considered. (2) The construction of a dynamic knowledge graph may help continuously optimize design and prediction models, such as incremental learning algorithms (online sequential extreme learning machine), online learning algorithms (adaptive linear neuron), and neural link prediction algorithms (neural collaborative filtering or matrix factorization techniques). This dynamic nature will allow the system to automatically absorb new data and knowledge to continuously optimize designs and predictive models. (3) Future research could explore approaches such as ensemble learning and deep learning to further improve prediction accuracy. (4) The real-time feedback and adaptive design are also important for the design process. By integrating machining learning models with real-time sensor data, an adaptive design process can be achieved where the model can automatically adjust design parameters based on new input data. This method’s application potential will be continually explored as sensor technology advances and application scenarios expand (including aerospace, medical devices, and smart manufacturing), and its value is expected to be realized in a broader range of fields in the future. 

## Figures and Tables

**Table 1:** The query is implemented in the strain sensor graph.

Code: Graph Query Implemented to Access Results
Query rules: Match (v:matrix{name: “PDMS”})\WHERE v. matrix.name == “PDMS”\ RETURN v;
Results = graph. run(match_str)

**Table 2:** Task 1 (Forward design to predict performance).

Input	Verification	
Flexible substrate: yarn	Gauge factor:50 Strain range: 90%	Reference [38]
Functional materials: rGO		
Method: dip-coating		
Flexible substrate: cotton	Gauge factor: 4 Strain range: 11.6%	Reference [39]
Functional materials: rGO		
Method: dip-coating		

**Table 3:** Task 2 (Inverse design to predict materials and structure).

Input	Verification	
Functional material: carbon nanotubes (CNTs)	Substrate: Flax fabric	Reference [40]
Gauge factor: 1.24		
Strain range: 120%		
Substrate: Polyaniline	Functional material: Silver nanowires	Reference [41]
Gauge factor: 1		
Strain range: 500%		
Strain range: 240%		

**Table 4:** The summarization of the reported sensor performance.

Ref.	Main Materials	Strain Range	Sensitivity	Repeatability	Response/Recovery Times	Limit Detection
[1]	CNTs ink/PU	350%	2.7	1000	~	~
[6]	CNTs/CNF/PDMS/TPU	217.5%	12.7	800	~	~
[7]	Silver fillers/LM ink	170%	~	5000		
[8]	CNTs/latex tube	200%	91.1	4000	290 ms/310 ms	0.1%
[15]	CB/PDMS	250%	12	1000	~	~
[16]	CNTs/CB/PDMS	80%	7.7	10,000	100 ms/110 ms	0.04%
[20]	CNTs/LiCl/elastic core-spun yarn	100%	1.35	1000	300 ms	~
[30]	Graphene/PU	160%	86.86	100	~	~
[31]	LM/TPU	548%	6	1000	~	~
[32]	CNTs/TPU	140%	~	1250	~	~
[33]	PAN/graphene/TPU	2%	1700	300	~	~
[37]	CNTs@carbon black/PDMS	80%	7.7	10,000		2%
[40]	CNTs/fabric	128%	4.73	~	~	~
[42]	MXene/TPU/PAN	80%	9.69	1750	140.6 ms	0.1%
[43]	Conductive ink/rubber	50%	12.14	5000	71.43 ms/178.49 ms	~
[44]	CNTs/cotton	300%	21.85	~	~	~
This work	Graphene/CB/TPU	300%	110	1000	110 ms/112 ms	~

## Data Availability

The original contributions presented in the study are included in the article/supplementary material, further inquiries can be directed to the corresponding authors.

## Supplementary Materials

The following supporting information can be downloaded at: https://www.mdpi.com/article/10.3390/s24175484/s1, Figure S1. The introduction of confusion matrix. Figure S2. The example of the partial knowledge graph. Figure S3. The cluster altas of different combinations of TranSE and DisMult methods. Table S1. The full names of materials. Table S2. The probability of combinations in the literature. Table S3. The coordinate of some functional materials and substrates, refs. [22,46,47,48,49,50,51,52,53,54,55,56,57,58,59,60,61,62,63].
