# Supplementary material for: Data-Driven Strain Sensor Design Based on a Knowledge Graph Framework
Source: Sensors (Basel). 2024 Aug 24;24(17):5484. doi: 10.3390/s24175484 (PMC11398124; doi:10.3390/s24175484)
Supplement: Supplementary file 1 [file sensors-24-05484-s001.zip › sensors-3147399-supplementary.pdf]

Supplementary Information

Data-Driven Strain Sensor Design Based on a Knowledge Graph Framework

Junmin Ke [1,2], Furong Liu [1,2,*], Guofeng Xu [1,2] and Ming Liu [1,2]

1 Key Laboratory of Trans-Scale Laser Manufacturing, Beijing University of Technology, Ministry of Education, Beijing 100124, China; liuming123@emails.bjut.edu.cn (M.L.)
2 School of Physics and Optoelectronic Engineering, Beijing University of Technology, Beijing 100124, China
* Correspondence: liufr@bjut.edu.cn

S1. Methods

In general, the representation learning process aims to extract local or global connectivity patterns between entities, and reasoning is performed by using these patterns to generalize the observed relationship between specific entities. Four representation learning methods are adopted to generate embedding, including the Node2vec, TranSE, DisMult and HOLE methods. As the final learning method, the HOLE method is adopted because it showed the best performance. After that, the multi-layer perception network (MLP) was designed and trained for the classification of reasoning combinations , predicting the feasibility of combinations. The details regarding the above methods are explained as follows:

Node2vec method

The Node2vec method seeks to encode each node into an embedding space while maintaining the network structure information. Encoding entity relationships allows us to capture the context of each data point in addition to its attributes. This algorithm is summarized as a two-part process. It begins by generating a sequence of nodes using biased second-order random walks. By fine-tuning random walk hyper-parameters, the performance of the model changes. Then, nodes serve as inputs for a skip-gram with a negative sampling model. The skip-gram model produces pairings of input and context nodes based on the size of the context window, before feeding them into a shallow neural network. We are able to obtain the hidden layer weights in the form of node embedding through the network training procedure. The size of an embedding is determined by the

number of hidden layer neurons. It is stated that similar nodes in the network should have similar embedding. We measure the similarity in the network between two nodes, and compare it to the similarity in the embedding space. Whenever the similarity in the embedding space between two nodes does not reflect the similarity in the network, the embedding is adjusted. In addition, the Node2vec method uses the cosine distance method to calculate the distance of the nodes.

TranSE and DisMult methods

The distance model comprises a training set S of triples (h,l,t) composed of two entities (h, t), and a relationship l. Relationships are represented as translations in the embedding space: if (h, l, t) holds, then the embedding of the tail entity t should be close to the embedding of the head entity h, plus a vector that depends on the relationship l, i.e., we want h + l≈ t when (h, l, t) holds (t should be a nearest neighbor of h + l), while h + l should be far away from t otherwise. In order to learn such an embedding, we choose the L_1 or L_2 norm. The equation can be expressed as follows:

$$\sum_{(h,l,t)\in S}\sum_{(h',l,t')\in S'}[\gamma + d(h+l,t) - d(h'+l,t')]_+ \quad (1)$$

where [x]+ denotes the positive part of x, and γ is a margin hyperparameter. The loss function (1) favors lower values of the energy for training triplets than for corrupted triplets.

Different from TranSE model, the DisMult method uses bilinear score function. It is a simplification of the neural tensor network. Given a triple S (e_1, r, e_2), it transforms the score function into the range (0,1) to obtain ground-truth confidence. The training objective is to minimize the margin-based ranking loss.

$$L(\Omega) = \sum_{(e_1',r,e_2')\in T (e_1',r,e_2')\in T'} \max\{S_{(e_1',r,e_2')} - S_{(e_1,r,e_2)} + 1,0\} \quad (2)$$

HOLE Method

Let $\theta \in \{e_i\}_{i=1}^{n_e} \cup \{r_k\}_{k=1}^{n_r}$ denote the embedding of a signal entity or relation; the score formula can be described as follows:

$$\Pr(\emptyset_p(s,o)=1|\theta) = \sigma(\eta_{spo}) = \sigma(r_P^T(e_s \circ e_o)) \quad (3)$$

let $f_{spo} = \sigma(r_p^T(e_s \star e_o))$, the gradient of eq. (3) is then given by:

$$\frac{\partial f_{spo}}{\partial \theta} = \frac{\partial f_{spo}}{\partial \eta_{spo}} \frac{\partial \eta_{spo}}{\partial \theta} \tag{4}$$

By iterating with stochastic gradient descent (SGD), the object's embedding is updated. This approach has two benefits: The first is the use of compositional representations, which enables information to be propagated between triples, global dependencies in data to be captured, and the desired relational learning effect to be learned. Another advantage is that the relationship can be modeled by correlation operation. Rather than simply storing associations, the HOLE model learns the embedding that best explains the observed data.

Support Vector Machines (SVMs)

This method constructs a hyper-plane in a high dimensional space for the classification. A good separation can generate the largest distance between the nearest training data and the hyper-plane.

XGBoost (XGB)

XGB can be understood as a parallel prediction of multiple trees, and the prediction scores are added, so that they can be judged. This tree model generates a new tree through continuous iteration, so that the predicted value can approach the true value.

Multi-layer Perception (MLP)

This method can learn a non-linear function for either classification or regression tasks, usually containing the input layer, hidden layer and output layer. For the classification, the loss function is given as

$$\mathrm{Loss}(\hat{y}, y, W) = -\frac{1}{n}\sum_{i=0}^{n}\left(y_i \ln \hat{y}_i + (1-y_i)\ln(1-\hat{y}_i)\right) + \frac{\partial}{2n}||W||_2^2 \tag{5}$$

And for the regression task, the loss function is

$$\mathrm{Loss}(\hat{y}, y, W) = -\frac{1}{n}\sum_{i=0}^{n}||\hat{y}-y||^2 + \frac{\partial}{2n}||W||_2^2 \tag{6}$$

S2. Evaluation Metrics

The multi-layer perception is adopted after the representation, outputting the label and probability of the combination. The activation function chooses the parameter of relu. The label has two values, indicating whether the combination is true or not. The output of 1 is true, while 0 is false. Meanwhile, the probability of the output denotes the chance of the label. In this method, the accuracy and precision are used for the evaluation indicator. The accuracy represents the proportion of the predicted correct quantity to the total quantity in positive and negative cases, while precision emphasizes the rate at which the correct category is correctly predicted. The overall accuracy of the classification task on the training dataset is 0.96, with an accuracy of 0.85 on the test dataset. The accuracy and precision are between 0 and 1. Moreover, higher accuracy and precision mean a better model. In addition, it is found that the probability of true combinations from the literature is between 0.74 and 0.99 (Table S3). Meanwhile, the false combinations are clearly less than 0.6. Considering this range of probability, the threshold used for identifying the feasibility of combinations is set as 0.74. The samples of probability are summarized in Table 2. Furthermore, the mean absolute error (MAE) and mean squared error are used for the regression task (XGB and MLP methods) of performance. In contrast to the accuracy, low relative values of MAE and MSE represent a good model.

Indicators	0	1
0	True negative (TN)	False negative (FN)
1	False positive (FP)	True Positive (TP)

Figure S1. The introduction of confusion matrix.

$$\text{Precision} = \frac{\text{TP}}{\text{TP} + \text{FP}} \tag{7}$$

$$\text{Recall} = \frac{\text{TP}}{\text{TP} + \text{FN}} \tag{8}$$

$$\text{Accuracy} = \frac{\text{TN} + \text{TP}}{\text{TN} + \text{FN} + \text{FP} + \text{TP}} \tag{9}$$

$$\text{F1} - \text{score} = \frac{2 \times \text{Pr}\,ecision \times \text{Re}\,call}{\text{Pr}\,ecision + recall} \tag{10}$$

$$\text{MSE} = \frac{1}{n}\sum_{i=1}^{n}(y_i - \hat{y}_i)^2 \tag{11}$$

$$\text{MAE}\left(y, \hat{y}\right) = \frac{1}{n}\sum_{i=0}^{n-1} | y_i - \hat{y}_i | \tag{12}$$

S2. Evaluation indicators of HOLE, TranSE and DisMult

Two evaluation indicators, including Mean Reciprocal Ranking (MRR) and Hit ratio, with the cut-off values n = 1, 3, 5, 10, are utilized. MRR is the average inverse rank for correct entities, with a higher value representing better performance (Figure. S2). Hit@n measures the percentage of correct entities in the top n predictions. The formula is represented as follows:

Mean Reciprocal Ranking (MRR):

$$\text{MRR} = \frac{1}{|S|}\sum_{i=1}^{|S|}\frac{1}{\text{rank}_i} = \frac{1}{|S|}\left(\frac{1}{\text{rank}_1} + \frac{1}{\text{rank}_2} + \ldots + \frac{1}{\text{rank}_{|S|}}\right) \tag{13}$$

Hit@n:

$$\text{HIT@n} = \frac{1}{|S|}\sum_{i=1}^{|S|}\prod(\text{rank}_i \le n) \tag{14}$$

The HOLE method has the highest value, up to 0.89, while the TranSE method has the lowest value (about 0.799). Although the algorithm of DistMult also has relatively higher values of MRR (~0.822), the vector-based calculation reduces its ability to capture information, further affecting accurate prediction.

S3 The scatter diagram of the HOLE without considering the relationship between the same property

We have found nine kinds of material combinations in the original representation method by the adjacent points. They are cotton and graphene, fabric and azo, ecoflex and Glycerol/kcl, ecoflex and kc/Gly, hydrogel and PAA/TA-CNCs, rubber and CNTs/PEDOT: PSS, cotton and graphene/cb, wool and graphene and PDMS/pt. Moreover, these above combinations are verified in the database.

Figures

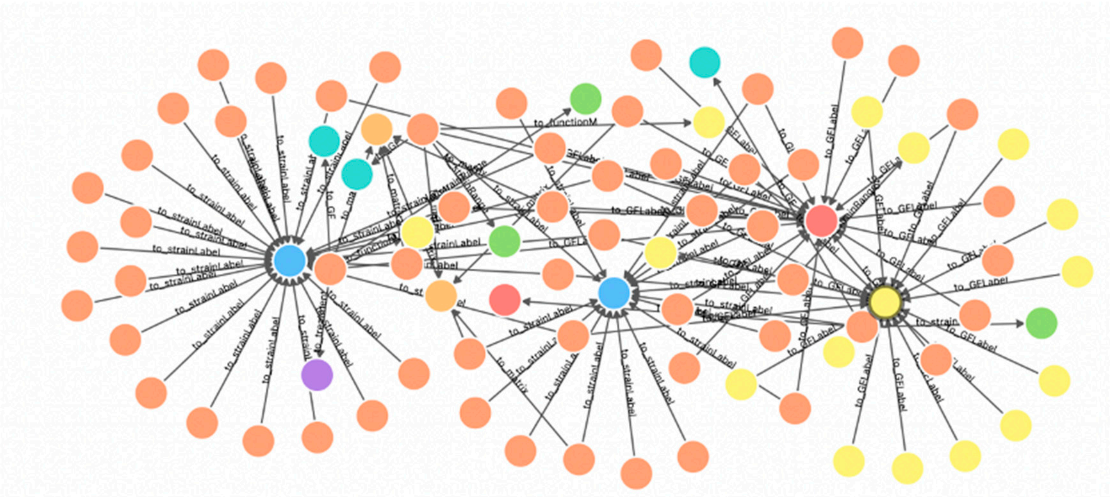

Figure S2. The example of the partial knowledge graph.

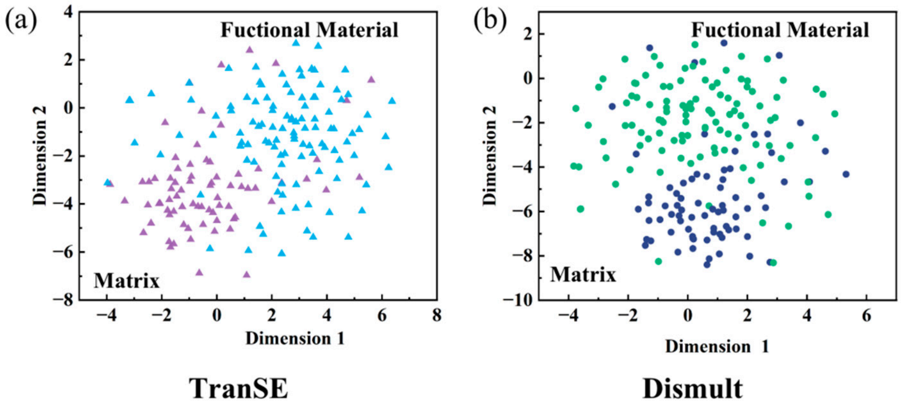

Figure S3. The cluster altas of different combinations of TranSE and DisMult methods.

Tables

Table S1. The full names of materials.

Index	Abbreviated name	Full name
1	rGO	reduced graphene oxide
2	CNTs	carbon nanotubes
3	MWCNTs	multi-wall carbon nanobutes
4	GO	graphene oxide
5	MXene	MXene (Ti3C2)
6	AgNWs	silver nanowires
7	SWNT	single-wall carbon nanobutes
8	ACNF	aligned carbon nanofiber
9	VN	anadium nitride nanoparticle
10	CB	carbon black
11	PANI	polyaniline
12	graphene	graphene
13	PMXene	polydopamine modified MXene
14	TPU	thermoplastic polyurethane
15	PI	polyimide
16	PTFE	polytetra fluoroethylene
17	Ecoflex	Ecoflex
18	PDMS	polydimethylsiloxane
19	PVDF	poly (vinylidene fluoride)
20	PVA	polyvinyl alcohol
21	AuNWs	au nanowires
22	SWCNT	single-wall carbon nanotubes
23	CNFs	cellulose nanofibrils
24	PPy	polypyrrole
25	PU	polyurethane
26	TPE	thermoplastic elastomer
27	AgNPs	silver nanoparticles
28	CNCs	cellulose nano-crystalline

Table S2. The probability of combinations in the literature.

Index	Combinations	Probability	Ref.
1	PDMS/CNTs/Mxene	0.84	[45]
2	Polyurethane/Mxene	0.78	[34]
3	TPU/CNTs	0.88	[46]
4	Fiber/MWCNTs	0.97	[47]
5	PDMS/MXene/poly(ta)	0.99	[48]
6	Fiber/graphene/pvdf	0.97	[49]
7	Paper/MXene	0.92	[50]
8	Yarn/cb/cncs	0.79	[51]
9	PDMS/CNTs	0.78	[52]
10	Nylon/PEDOT: PSS/AgNWs	0.87	[53]
11	Polyimide/MXene/TiO2	0.92	[54]
12	PDMS/rGO	0.74	[55]
13	Rubber/CNTs	0.78	[56]
14	polyvinyl alcohol/cnfs/znso4	0.97	[57]
15	Ecoflex/egain alloy/sio2 microspheres	0.96	[58]

Table S3. The coordinate of some functional materials and substrates.

Functional material	Flexible matrix	Coordinate of the functional material	Coordinate of the flexible matrix	Distance	Ref
Glycerol/ kcl	ecoflex	(4.38024, 3.37265)	(3.58242, 3.64646)	0.714	[58]
MXene/ CNCs	polyurethane	(1.04869, -2.493)	(-2.601, -6.121)	26.41	[59]
CNTs/ MXene	rubber	(-1.83273, - 5.20404)	(-1.74536, - 4.57424)	0.507	[60]
CB/ MWCNTs	PDMS	(3.64621, 7.4754)	(3.45073, 7.39312)	0.046	[61]
pedot:pss/ agnws	nylon	(-0.41206, 3.89873)	(0.00308, 4.10123)	0.212	[62]
Graphene nanoribbons	TPU	(4.46887, -2.55121)	(2.63917, - 0.51695)	7.432	[63]
MXene/ CNTs	TPU	(3.72447, -0.73028)	(2.63917, - 0.51695)	1.221	[2]
Graphene/ cb	TPU	(1.42992, 4.40013)	(2.63917, - 0.51695)	25.45	This work

The distance of the above combinations in the improved representation method is less than 26.41. Therefore, the threshold of the matrix and functional material (D < 26.41) in the embedding space is determined in the present study to help evaluate the possibility of combinations for strain sensor designs. The distance of the discovered design is also found within this range.
